# Heterozygous eNOS Deficient Mice as a Model to Examine the Effects of eNOS Haploinsufficiency on the Cerebral Circulation

**Published:** 2017-02-07

**Authors:** Sean P. Didion

**Affiliations:** Department of Pharmacology and Department of Neurology, The University of Mississippi Medical Center, USA

**Keywords:** Endothelial nitric oxide synthase, NOS3, Single nucleotide polymorphisms, Mouse model, Cerebrovascular disease

## Abstract

Nitric oxide derived from endothelial nitric oxide synthase (eNOS) has been shown to be a major mediator of endothelium-dependent responses in cerebral blood vessels. Loss of a single eNOS gene is not associated with any apparent negative consequences on endothelial function in most blood vessels. In contrast, we have recently demonstrated that heterozygous eNOS gene deficiency in combination with a high fat diet is associated with marked impairment of endothelial function. These findings provide an important example of eNOS haploinsufficiency and one that directly impacts the cerebral vasculature. A major mechanism associated with the impairment of endothelial function with eNOS deficiency and a high fat diet appears to be related to increases in plasma IL-6 that serves to further reduce the bioavailability of NO either directly or indirectly via reductions in eNOS expression or activity and via increases in vascular superoxide. Taken together, these findings provide important insights into genetic and molecular mechanisms that promote endothelial dysfunction in response to a high fat diet in cerebral blood vessels with inherent reductions in eNOS gene expression, such as those due to eNOS gene polymorphisms. These findings also highlight the importance of eNOS+/− mice to study the effects of eNOS haploinsufficiency on cerebral blood vessels.

Endothelium-dependent responses of cerebral blood vessels are mediated in large part by nitric oxide derived from endothelial nitric oxide synthase (eNOS). For example, endothelium-dependent relaxation to agonists, such as acetylcholine, or changes in blood flow can be significantly reduced by pharmacological inhibition of NOS or genetic deletion of the eNOS gene^1–6^. While there appears to be some compensation for the loss of eNOS gene expression by neuronal NOS in cerebral arterioles there does not appear to be compensation for the loss of eNOS in the carotid artery, suggesting that endothelium-dependent responses in the carotid are entirely mediated by eNOS^1,2,4^.

eNOS expression and activity is regulated at both the transcriptional and translational levels as well as by post-translational mechanisms including phosphorylation, acetylation and sumolyation^7^. Alterations in eNOS cofactor bioavailability (i.e., increases in heat shock protein 90 (Hsp90) expression and reductions in L-arginine or tetrahydrobiopterin) can also result in reduced eNOS activity and thus negatively impact endothelial responses in cerebral blood vessels^7^. Once generated, NO bioavailability can be limited by increases in vascular superoxide. For example, the reaction of superoxide with nitric oxide produces peroxynitrite, which is a more oxidative molecule involved in nitration of proteins and DNA^8,9^. Superoxide-mediated inactivation of NO is a major mechanism of endothelial dysfunction in many diseases, such as atherosclerosis, diabetes, hypertension, and obesity^10,11^. Examination of vascular responses of cerebral blood vessels is clinically important, as endothelial dysfunction has emerged as a strong independent predictor of future cerebrovascular disease and events, such as carotid artery disease and stroke^12,13^.

We, and others, have shown that heterozygous eNOS gene deficiency is associated with normal responses to endothelium-dependent agonists, such as acetylcholine, in the aorta and carotid artery^6,14^. The fact that the response to acetylcholine is normal is surprising considering that heterozygous eNOS deficiency is associated with a 50–60% reduction in eNOS protein^14^. We recently demonstrated that the majority of the response to acetylcholine in carotid arteries from *eNOS^+/−^* mice can be inhibited by L-NNA (a NOS inhibitor) and ODQ (an inhibitor of sGC)^14^. In contrast, responses to nitroprusside are almost completely inhibited by ODQ^14^. These findings provide pharmacological evidence that responses of carotid artery from *eNOS^+/−^* mice to acetylcholine and nitroprusside, like those in wild-type *eNOS^+/+^* mice, remain almost exclusively dependent on NOS and sGC and sGC, respectively. These data also suggest that there must be some compensatory mechanism(s), which serves to maintain a normal degree of endothelial function in the absence of a single eNOS gene.

At least two potential mechanisms have been described to account for normal vascular responses associated with a single eNOS gene. The first mechanism involves increased soluble guanylyl cyclase (sGC) sensitivity^4,15^. sGC serves as the “NO receptor” in vascular muscle^8^. NO binding to sGC results in increases in intracellular cGMP formation and subsequent relaxation of vascular muscle through a protein kinase G-dependent mechanism^8^. Indeed, several laboratories have demonstrated that responses of carotid artery from *eNOS^−/−^* and *eNOS^+/−^* mice to NO donors, such as nitroprusside, are greater than those in wild-type (*eNOS^+/+^*) mice, suggesting that the sensitivity to NO increases in the absence of eNOS^4,15^. The increased relaxation that occurs in response to NO donors is greater in the absence of two eNOS genes compared to that in the absence of a single eNOS gene, suggestive of a gene dosing effect. Moreover, the increase in sensitivity to NO appears to be related to compensatory increases in sGC activity per se rather than increases in expression of the alpha or beta subunits of sGC^15^.

A second mechanism that could also account for the normal responses to acetylcholine in *eNOS^+/−^* mice appears to involve increased activity of eNOS itself. We recently demonstrated that despite the marked reduction in eNOS protein in *eNOS^+/−^* mice there appears to be a compensatory increase in eNOS phosphorylation at Ser1176 (equivalent to Ser1177 and Ser1179 in human and bovine eNOS respectively)^14^, an eNOS phosphorylation site associated with enhanced eNOS activity as well as increased levels of NO^7^. An increase in Ser1176 phosphorylation would most likely be reflective of increases in upstream activity of protein kinase A and protein kinase B (Akt), two kinases that have been shown to phosphorylate Ser1176, decreases in dephosphorylation of Ser1176 due to reductions in PP2A expression or activity, as well as alterations in caveolin-1 and Hsp90, both important regulators of eNOS activity^10,11^. Additional studies are required to further define the specific molecular events that serve to maintain endothelial function in lieu of the normal complement of eNOS protein.

Although a large amount of information regarding the role of eNOS in vascular responses has been obtained from *eNOS^−/−^* mice such studies do not appear to have a human correlate, as there is no evidence that homozygous eNOS deficiency occurs in the human population. While complete absence of eNOS may not occur in humans, a number of genetic polymorphisms have been identified in the human eNOS gene including in the promoter region (T-1468A; A-922G; T-786C), introns (Intron 4 VNTR; Intron 23 G10T), and exons (Exon7, G894T)^16–21^. Perhaps the best-studied eNOS polymorphism is the T-786C polymorphism, which is associated with reductions in eNOS gene expression as well as serum NO levels. The T-786C polymorphism is associated with increased risk of preeclampsia, coronary artery disease, and hypertension^16–21^. Surprisingly, much less is known regarding the association of the T-786C eNOS polymorphism and cerebral vascular function or disease. Thus, future studies will be extremely important in identifying those patients that may be at higher risk for cerebral vascular dysfunction and disease. Indeed, a few studies have begun to link the T-786C eNOS polymorphism with increased risk for intracranial hemorrhage^22–24^. While studies of vascular function in human cerebral vessels is currently limited, future studies in humanized mice or rats that express human eNOS gene polymorphisms will be particularly invaluable in studying the direct impact of specific eNOS polymorphisms on cerebral vascular function alone or in combination with other cardiovascular risk factors, such as aging and hypertension.

In lieu of humanized mouse models of eNOS polymorphisms, we propose that the *eNOS^+/−^* mouse is an excellent model to study the effects of inherent reductions in eNOS gene expression on responses of cerebral blood vessels. The *eNOS^+/−^* mouse with its 50–60% reduction in eNOS expression closely mimics the 50% reduction in eNOS expression associated with the T-786C eNOS polymorphism^21^. We have recently demonstrated, using *eNOS^+/−^* mice, that heterozygous eNOS deficiency in combination with 30 wks of a high fat diet (a time point not normally associated with endothelial dysfunction in wildtype *eNOS^+/+^* mice) is associated with marked impairment of endothelial function in the carotid artery^14,25^. A high fat diet is closely associated with increases in plasma interleukin-6, a pro-inflammatory cytokine that has been shown to produce marked reductions in eNOS protein expression (in a dose-dependent fashion) as well as increases in vascular superoxide^26,27^.

Moreover, in proof-of-concept experiments, incubation of carotid arteries with recombinant IL-6 had no effect on vascular responses in vessels from wild-type *eNOS^+/+^* mice^14^. In contrast, IL-6 produced marked impairment of endothelium-dependent responses in carotid artery from *eNOS^+/−^* mice^14^. The impairment of endothelial responses by IL-6 was accompanied by increases in NADPH oxidase-derived superoxide. These data serve to link the increase in plasma IL-6 in *eNOS^+/−^* mice in response to a high fat diet and the impairment of endothelial function. These data provide an important example of eNOS haploinsufficiency that may have important clinical implications in terms of endothelial function and cerebrovascular risk in obese patients. Future studies in humans will be necessary to determine if eNOS polymorphisms alone or in combination with other cardiovascular risk factors predispose individuals to cerebral vascular disease and events, such as carotid artery disease and stroke.

In conclusion, loss of a single eNOS, while insufficient to impact endothelial function under baseline conditions, predisposes cerebral blood vessels to endothelial dysfunction in response to a high fat diet. Such findings serve as an important example of eNOS haploinsufficiency and may be predictive of greater cerebrovascular risk in obese patients with polymorphisms in the eNOS gene. While a number of eNOS polymorphisms have been linked to coronary artery disease and risk, much less is known regarding the impact of eNOS gene polymorphisms on the cerebral blood vessels and cerebrovascular disease^28,29^. We highlight an example of eNOS haploinsufficiency that occurs in the carotid artery in *eNOS^+/−^* mice fed a high fat diet. Based on these findings *eNOS^+/−^* mice appear to be an excellent model and one, which mimics the effect of eNOS polymorphisms in the eNOS promoter, such as those associated with reductions in eNOS protein expression. Future studies employing *eNOS^+/−^* mice will be critical to further our understanding of the specific cellular and molecular mechanisms that contribute to vascular dysfunction in cerebral blood vessels.
